# Chemical Composition and Antioxidant, Analgesic, and Anti-Inflammatory Effects of Methanolic Extract of *Euphorbia retusa* in Mice

**DOI:** 10.1155/2018/4838413

**Published:** 2018-07-04

**Authors:** Jazia Sdayria, Ilhem Rjeibi, Anouar Feriani, Sana Ncib, Wided Bouguerra, Najla Hfaiedh, Abdelfattah Elfeki, Mohamed Salah Allagui

**Affiliations:** ^1^Research Unit of Macromolecular Biochemistry and Genetic, Faculty of Sciences of Gafsa, University of Gafsa, 2112 Gafsa, Tunisia; ^2^Laboratory of Animal Ecophysiology, Faculty of Science of Sfax, University of Sfax, 3018 Sfax, Tunisia; ^3^Common Services Unit for Research, Faculty of Sciences of Gafsa, University of Gafsa, 2112 Gafsa, Tunisia

## Abstract

Plants provide an alternative source to manage different human disorders due to various metabolites. The aim of this study is to investigate the phytochemical constituents of the methanolic extracts of *Euphorbia retusa* and to evaluate their antioxidant, anti-inflammatory, and analgesic activities. The phytochemical results obtained by HPLC and by chemical assay reactions have revealed the richness of the methanolic extract of *E. retusa* in active compounds, in particular polyphenols, flavonoids, and tannins. The methanolic extract shows significant antioxidant activities *in vitro*, in the DPPH and the FRAP assays. The antinociceptive activity was evaluated using acetic acid and hot-plate models of pain in mice. The anti-inflammatory activity was evaluated by carrageenan-induced paw edema. Oral pretreatment with the methanolic extract of *E. retusa* (200 mg/kg) exhibited a significant inhibition of pain induced either by acetic acid or by the heating plate and in a manner comparable to the standard drug paracetamol. *E. retusa* significantly reduced paw edema starting from the 3rd hour after carrageenan administration by increasing the activity of antioxidant enzymes (SOD, CAT, and GPx) in liver and paw tissues and decreasing the levels of MDA. These results may confirm the interesting potential of this plant as a treatment of various inflammatory and pain diseases.

## 1. Introduction

Inflammatory reaction is one of the most important defense mechanisms against tissue injury caused by physical and chemical agents, noxious stimuli, heat, antigen-antibody reaction, and microbial effect. Inflammatory response occurs in two distinct phases: an acute and a chronic. The acute phase is a preliminary crisis absolutely necessary for the healing processes characterized by local vasodilatation, increased capillary permeability, and the release of inflammatory mediators like histamine, serotonin, and prostaglandins. The chronic phase is the result of failure to eliminate acute inflammation, an autoimmune response to a self-antigen characterized by infiltration of leukocytes and phagocytic cells [1]. Chronic inflammation can last for several months and even years and can eventually cause several diseases such as rheumatoid arthritis, atherosclerosis, asthma, heart disease, ulcerative colitis, and some cancers. Therefore, inflammation must be well regulated in these early stages.

Several studies have demonstrated that the main mechanisms involved in the inflammatory reaction are closely associated with the generation of free radicals and the creation of oxidative stress. In fact, during the inflammation process, several reactive oxygen species (ROS) including nonradical hydrogen peroxide (H_2_O_2_), singlet oxygen (^1^O_2_), nitric oxide (NO), superoxide (O2^·−^), hydroxyl (HO^·^), and peroxyl (ROO^_^) radicals are overexpressed by neutrophils and macrophages and play an important role in the host defense mechanism [2]. Besides their defensive effects, this overproduction leads to tissue injury by damaging macromolecules and lipid peroxidation of membranes [3]. In addition, ROS propagate inflammation by stimulating the release of cytokines, such as interleukin-1, tumour necrosis factor-alpha (TNF-*α*), and interferon-gamma (IFN-*γ*). Thus, free radicals are important mediators that provoke or sustain inflammatory processes, and consequently, cells and tissues require antioxidant enzymes, including superoxide dismutase, catalase, and glutathione peroxidase to neutralize and decompose these reactive oxygen species [2, 4].

Currently, nonsteroidal anti-inflammatory drugs (NSAIDs) are widely prescribed because of their efficacy in the management of pain, inflammation, and rheumatic disorders. However, their long-term therapeutic use is often associated with adverse effects such as gastrointestinal ulcers and renal insufficiency [5]. For these reasons, the use of medicinal plants has become the new strategy of several studies to develop and introduce new drugs with greater safety and efficiency. Indeed, medicinal plants contain a large number of bioactive molecules of multiple interests not only in traditional medicine but also in the food and pharmaceutical industry. These molecules include alkaloids, phenolic acids, tannins, and flavonoids which possess interesting biological activities like anti-inflammatory, anticarcinogenic, antimicrobial, and antioxidant.

*Euphorbia* is the largest genus of the family Euphorbiaceae and comprises about 1000 species. Many researchers have shown that *Euphorbia* species possess antitumoral activity [6], antimicrobial activity [7], inhibition of HIV-1 viral infection [8], and antihyperglycemic and hypolipidemic activities [9]. *Euphorbia retusa* locally called “lebina” is an annual herb abundantly found throughout the Mediterranean area. It is characterized by long alternate blue-green leaves which contain toxic and skin-irritant milky latex very rich in tetracyclic triterpene compounds [10]. This herb has been used in traditional medicine as a cure for warts, trichiasis, and venomous bites [11]. However, to our knowledge, *E. retusa* is less studied than the other species of their family. There is some scientific work that has reported the isolation of many compounds including flavonol glycosides [12] and triterpenoids and fatty acids from the aerial part of this plant [13]. Diterpenoids and triterpenoids were also isolated from the roots [13]. For biological properties, only a recent study conducted by Abdallah [14] has demonstrated the antimicrobial activity of this plant. Therefore, the present study is aimed at determining the phytochemical composition of the methanolic extract of *E. retusa* using standard analytical methods followed by exploring their potential antioxidant, antinociceptive, and anti-inflammatory activities by *in vivo* and *in vitro* models.

## 2. Materials and Methods

### 2.1. Chemicals and Reagents

The chemicals and reagents used in this work such as Folin–Ciocalteu reagent, 2,2-diphenyl-1-picrylhydrazyl (DPPH), ascorbic acid, gallic acid, vanillic acid, rutin, epicatechin, coumaric acid, catechin, 2,6-di-tert-butyl-4-hydroxy-carboxylic acid (BHT), sodium carbonate, aluminum chloride (AlCl_3_), sodium nitrate (NaNO_2_), sodium carbonate (Na_2_CO_3_), sodium hydroxide (NaOH), and hydrogen peroxide (H_2_O_2_) were purchased from Sigma-Aldrich Chemical (St. Louis, MO). HPLC-grade solvent methanol, acetonitrile, and acetic acid were purchased from Carlo Erba Reactif-CDS. Carrageenan was also obtained from Sigma-Aldrich Chemical (St. Louis, MO).

### 2.2. Plant Material

The leaves of *Euphorbia retusa* were collected from the Gafsa region in the southwest of Tunisia (34°22′59″N latitude and 8°09′00″E longitude) during November 2015. The plant leaves were washed and dried in shade for up to two weeks. The dried leaves were crushed, using an electric grinder, into fine powders and stored at room temperature in a dry place.

### 2.3. Extraction Procedure

The dried powder leaves of *E. retusa* were extracted by the maceration method. 50 mL of methanol was added to 5g of leaves and was left to stand under agitation for 24 h. Subsequently, the solution was filtered using a filter paper. The methanol extract was evaporated under reduced pressure at room temperature, and the obtained residue was preserved at 4°C for further analysis.

### 2.4. Determination of Total Polyphenol Contents

The total polyphenol content of the methanol extract was carried out according to the Folin–Ciocalteu method [15]. This method consists of mixing 0.25 mL of the extract (1 mg/mL) with 1.25 mL of the Folin–Ciocalteu reagent (diluted 10 times with distilled water). After 5 min, 1 mL of sodium carbonate solution (7.5%) was added, and the mixture was incubated at 45°C for 2 hours. The absorbance of the solution obtained was measured at 765 nm using a spectrophotometer (Shimadzu UV-190). A calibration curve was prepared in parallel under the same operating conditions using gallic acid as positive control. The phenol contents were expressed in terms of milligrams of the gallic acid equivalent (GAE) per gram of the extract (mg·GAE/g extract).

### 2.5. Determination of Flavonoid Contents

The flavonoid content was determined according to the aluminum chloride colorimetric method of Djeridane et al. [16]. Briefly, 0.5 mL of the extract (1 mg/mL) was mixed with 0.15 mL of 5% sodium nitrite. After 5 min, 0.15 mL of 10% AlCl_3_ solution was added to the mixture, followed by the addition of 1 mL of 1.0 M·NaOH after one min, and diluted with 1.2 mL of distilled water. The standard solution of quercetin (0–400 *μg*/mL) was prepared in the same conditions. The absorbance of the extract and standard solutions was read at 430 nm using a spectrophotometer (Shimadzu UV-190). The total flavonoid content was determined from the calibration curve and expressed as milligrams of the quercetin equivalent (QE) per gram of the extract (mg·QE/g extract).

### 2.6. Condensed Tannin Contents

The Broadhurst and Jones method [17] modified by Heimler et al. [18] was used for the determination of the condensed tannin content. 400 *µ*L of the extract (1 mg/mL) or standard solution (1 mg/mL) was mixed with 3 mL of 4% vanillin initially dissolved in methanol and 1.5 mL of concentrated sulfuric acid. The mixture was incubated for 15 min, and the absorbance was taken at 430 nm using a spectrophotometer (Shimadzu UV-190). Catechin solution was used as a reference, and the content of condensed tannins was expressed as mg of the catechin equivalent/mg of the extract.

### 2.7. Identification of the Phenolic Compounds and Flavonoids by HPLC

For the identification of the phenolic compounds and flavonoids present in the plant extract, a Varian ProStar HPLC system equipped with a reverse C18 column (Varian; 150 nm × 4.6 mm, particle size 5 *μ*m), a ternary pump (ProStar 230), and a diode barrier detector (ProStar 330) was used. The chromatographic analyses were a gradient elution consisting of two solvents: solvent A (100% methanol) and solvent B (0.05% acetic acid aqueous solution). The gradient conditions were 35% A and 65% B at *t* = 1 min, 50% A and 50% B at *t* = 30 min, and 90% A and 10% B at *t* = 40 min. The flow rate was 1 mL/min, and the injection volume was 20 *µ*L at 25°C. The identification was carried out at 280 nm for phenolic acids and at 365 nm for flavonoids using gallic acid, epicatechin, coumaric acid, apigenin, and naringenin as standard phenolic acids and rutin, quercetin, and kaempferol as standard flavonoids and based on comparison with retention times and coinjection.

### 2.8. Antioxidant Activity

#### 2.8.1. DPPH Radical Scavenging Activity Assay

The capacity to scavenge the free radical 2,2-diphenyl-1-picrylhydrazyl (DPPH) was estimated according to the method of Yang et al. [19]. Briefly, 1 mL of the methanol extract at different concentrations (0.05 to 0.4 mg/mL) was mixed with 1 mL of 50 mm DPPH ethanolic solution. The mixtures were kept in the dark for 30 min, and the absorbance was measured at 517 nm using Shimadzu UV-190. Butylhydroxytoluene (BHT) used as positive control was prepared under the same conditions. Each test was performed in triplicate. The DPPH radical scavenging activity (RSA) was calculated as follows:

(1) $$\begin{array}{c} \text{RSA }\left(\%\right)=\frac{\text{absorbance of DPPH}-\text{absorbance of the sample}}{\text{absorbance of DPPH}}\times 100. \end{array}$$

#### 2.8.2. Ferric-Reducing Antioxidant Power Assay

The ability of the extract to reduce the ferric ion (Fe^3+^) present in the K_3_[Fe(CN)6] complex to ferrous ion (Fe^2+^) was evaluated by the method described by Oyaizu [20]. Briefly, 2.5 mL of the plant extract at different concentrations (0–300 *µ*g/mL), 2.5 mL of sodium phosphate buffer (0.2 M, pH 6.6), and 2.5 mL of potassium ferricyanide (1% w/v in distilled water) were combined in a test tube and incubated in a water bath at 50°C for 20 min. Following this, 2.5 mL of trichloroacetic acid solution (10%, w/v) was added, and the mixture was centrifuged at 3000 rpm for 10 min. Finally, a 2.5 mL aliquot of the supernatant was mixed with 2.5 mL of distilled water and 0.5 mL of a 0.1% (w/v) solution of ferric chloride (FeCl_3_). The manipulation was carried out in triplicate. The absorbance was read spectrophotometrically at 700 nm. Ascorbic acid was used as a standard antioxidant, and the results were presented as the change in absorbance as a function of concentration; increased absorbance of the reaction mixture indicates greater reducing power.

### 2.9. Pharmacological Activities *In Vivo*

#### 2.9.1. Animals

Swiss albino mice weighing 20–30 g were obtained from the Central Pharmacy of Tunisia, Tunis, Tunisia. The animals were housed in a spacious indoor cage and were kept in the animal room under controlled conditions (12 h light-dark cycle; temperature 22 ± 2°C; relative humidity 60 ± 5%) with free access to food pellets (SNA, Sfax, Tunisia) and tap water ad libitum. Before two weeks of the experiment, the animals were divided into normal control groups and test groups pretreated with the methanolic extract of *E. retusa*, paracetamol, and indomethacin of six animals per group. The experiments on laboratory animals were carried out according to Guide for the Care and Use of laboratory Animals approved by the Animal Ethics Committee.

#### 2.9.2. Acute Toxicity

For evaluating the acute toxicity, mice were divided into different groups and treated with increasing oral doses of the *E. retusa* methanolic extract (100–200–400–600–800–1000 mg/kg) in distilled water. After administration of the extracts, the animals were kept under regular observation for 48 h for any mortality or behavioral changes.

#### 2.9.3. Analgesic Activity

*(1) Hot-Plate Test*. This test was carried out according to the method described by Turner [21]. It is based upon induction of thermal stimulus by putting the mice in a glass beaker setting on the surface of the hot plate thermostatically controlled at 55°C. Reaction time, measured in seconds, was recorded when the animals licked their forepaws and jumped. The increase in reaction time indicates analgesic activity. For this test, 3 groups of 6 mice each were used. Group 1 (control group) received 1 mL distilled water, group 2 (*E. retusa* group) was pretreated orally (p.o.) with the *E. retusa* methanol extract (200 mg/kg), and group 3 (positive control group) was pretreated with paracetamol (100 mg/kg, p.o.). *E. retusa* extract and paracetamol were administered with free access to an aqueous solution of both for two weeks.

*(2) Writhing Test*. The analgesic effect of the plant extract was assessed by the acetic acid abdominal constriction test according to the method described by Sawadogo et al. [22]. The animals were selected and divided into three groups of six mice each. Group 1 served as a normal control and was given 1 mL distilled water. Group 2 was pretreated orally (p.o.) with the *E. retusa* methanol extract at 200 mg/kg. Group 3 was pretreated with standard paracetamol at 100 mg/kg, p.o. After 15 days of pretreatment, all mice were intraperitoneally injected with 10 mL of 1% acetic acid. The number of abdominal writhings for each mouse was recorded during 30 min. Analgesic activity was expressed as a percentage of pain inhibition which was calculated according to the following formula:

(2) $$\begin{array}{c} \%\text{ inhibition}=\frac{\left(\left(\text{number of writhes}\right)\text{ control}-\left(\text{number of writhes}\right)\text{ treated}\right)}{\left(\text{number of writhes}\right)\text{ control}}\times 100. \end{array}$$

#### 2.9.4. Anti-Inflammatory Activity

The anti-inflammatory activity of *E. retusa* was evaluated using the carrageenan-induced mice paw edema model [23]. Inflammation was induced by the injection of 1% carrageenan in physiological water (0.9% NaCl) at a dose of 100 *μ*L into the subplantar region of the right hind paw of the mice. Four groups of six mice per group were used for the study: an untreated negative control group receiving a physiological solution by subplantar injection, an untreated positive control group injected with carrageenan (Carr), a reference group pretreated orally with indomethacin (10 mg/kg, p.o.) and injected with carrageenan (Carr + Indo), and the fourth group pretreated orally with the *Euphorbia* methanol extract (200 mg/kg, p.o.) and injected with carrageenan (Carr + Eu). The evolution of the volume of edema was followed by the measurement of the paw diameter of each mouse before and at 1, 2, 3, 4, 5, and 6 h after induction of inflammation using a manual calliper. For each group, the percentages of inflammation and inhibition of edema were calculated by the following formulas:

(3) $$\begin{array}{c} \%\text{ inflammation}=\frac{\left(\text{V}T-\text{V0}\right)}{\text{V0}}\times 100,\\ \%\text{ inhibition}=\frac{\left(\left(\text{V}T-\text{V0}\right)\text{ control}-\left(\text{V}T-\text{V0}\right)\text{ treated group}\right)}{\left(\text{V}T-\text{V0}\right)\text{ control}}\times 100, \end{array}$$

where V*T* is the paw size obtained at various times, while V0 is the paw size obtained before any treatment.

At the end of the experiment, the animals were sacrificed and the right hind paw tissue and liver were dissected, rinsed in ice-cold normal saline, and homogenized in ice-cold phosphate buffer (pH 7.4). Then, the homogenate was centrifuged at 3000 × g for 10 min. The supernatant was collected and kept at −20°C in a refrigerator for MDA and the antioxidant enzymes (CAT, SOD, and GPx) activity assays.

*(1) Determination of Tissue Lipid Peroxidation*. The concentration of malondialdehyde (MDA), an indicator of lipid peroxidation, was evaluated by the thiobarbituric acid-reacting substances (TBARS) method [24]. This method is based on the fact that peroxidation of most membrane systems leads to the release of small amounts of MDA which react with thiobarbituric acid (TBA) in the acidic high temperature and form a red-complex TBARS. The absorbance of TBARS was determined at 532 nm. Lipid peroxidation was expressed as nmoles of TBARS.

*(2) Determination of Antioxidant Enzyme Activity*. The biochemical parameters were analyzed to check the protective activity of the methanolic extract of *E. retusa* by the following methods: total superoxide dismutase (SOD) activity was evaluated by measuring its ability to inhibit the photoreduction of nitroblue tetrazolium (NBT) [25].The photoreduction of NBT was mediated by superoxide anions generated by the xanthine/xanthine oxidase system and monitored at 550 nm. One unit of SOD is defined as the amount of enzymes required to inhibit the rate of NBT reduction by 50%. Total catalase (CAT) activity estimation was determined by measuring the hydrogen peroxide dissipation at 240 nm, using the method of Aebi [26]. Glutathione-peroxidase (GPX) activity was measured by detecting the oxidating state of NADPH at 340 nm according to the method used by Flohe and Gunzler [27]. The protein concentration of the tissue was determined by the method of Bradford [28], using bovine serum albumin as a standard.

### 2.10. Statistical Analysis

Results in this study were presented as the mean ± standard deviation (SD) and as the percentage. All analyses were statistically evaluated by ANOVA and using Student's *t*-test. *p* ≤ 0.05 was considered significant.

## 3. Results

### 3.1. Total Phenolic, Flavanoid, and Condensed Tannin Content

The total polyphenolic content of the leaf extracts was approximately 280.21 ± 0.085 mg of gallic acid/g of plant extracts. The total flavonoid content was estimated to be equivalent to 20.50 ± 0.107 mg of quercetin/g of plant extracts. Besides, the condensed tannin content of the leaf extracts of *E. retusa* expressed in mg of the catechin equivalents/g of plant extracts was 41.39 ± 0.198 mg/g (Table 1).

### 3.2. HPLC Analysis

**Figure 1:**
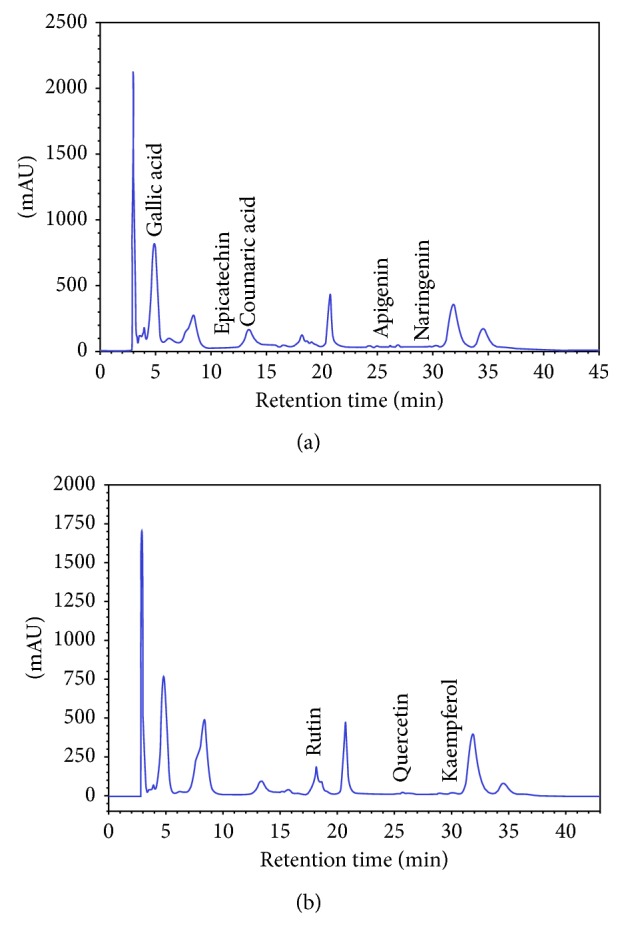
HPLC/DAD chromatogram of the methanol extract of *Euphorbia retusa*. (a) HPLC profile of phenolic acids at 280 nm; (b) HPLC profile of flavonoids at 360 nm.

The HPLC analysis of the *E. retusa* methanol extract revealed the presence of phenolic acids and flavonoids. The HPLC elution profile recorded at 280 nm showed 5 phenolic acids: gallic acid, epicatechin, coumaric acid, apigenin, and naringenin (Figure 1(a)) with concentrations of 1111.41 *μ*g/mL, 198.52 *μ*g/mL, 147.73 *μ*g/mL, 7.89 *μ*g/mL, and 6.13 *μ*g/mL, respectively (Table 2), while the HPLC elution profile of flavonoids (Figure 1(b); Table 2) recorded at 360 nm identified 3 compounds: rutin, quercetin, and kaempferol, with concentrations of 50.74 *μ*g/mL, 6.78 *μ*g/mL, and 14.42 *μ*g/mL.

### 3.3. Antioxidant Capacity

#### 3.3.1. DPPH Radical Scavenging Activity

**Figure 2:**
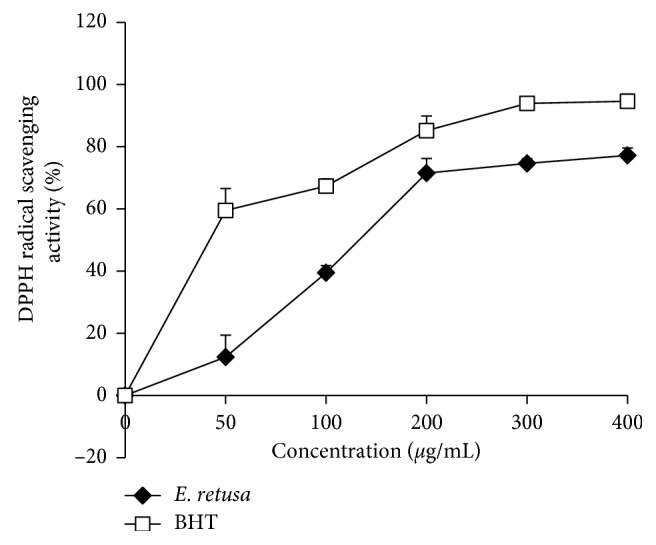
The antiradical activity of *Euphorbia retusa* against the radical DPPH. Values are represented as mean ± standard deviation (*n*=3).

The evolution of the antiradical activity of the extract of *Euphorbia*, *in vitro*, against the DPPH radical was dose-dependent (Figure 2). Indeed, as the concentration of the extract increases, the anti-DPPH activity also increases until a maximum concentration (0.4 mg/mL) was reached. The antioxidant capacity was determined from IC_50_, which corresponds to the concentration necessary to reduce 50% of the DPPH radical. The extract of *E. retusa* presents IC_50_ with 130 mg/mL which means low activity compared to BHT used as a positive control (40 mg/mL) (Table 1)

#### 3.3.2. Ferric-Reducing Antioxidant Assay (FRAP)

**Figure 3:**
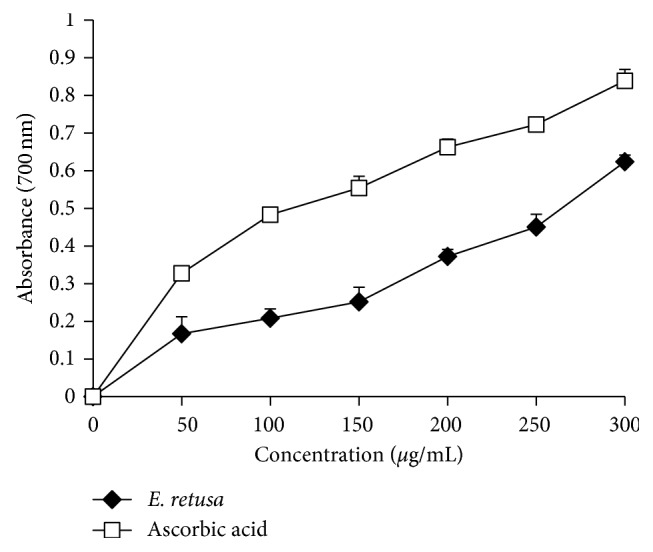
The reducing power of *Euphorbia retusa* and ascorbic acid by the FRAP assay. Values are expressed as mean ± standard deviation (*n*=3).

The antioxidant capacity of the extract was also measured by the FRAP method. As shown in Figure 3, the reducing power of the extract was dependent on the extract concentration compared to the ascorbic acid standard. The activity increased with the increasing concentration of the extract. The reducing power of the *Euphorbia* extract at 300 mg/mL was found to be 0.61 ± 0.12 of the absorbance unit. This activity appeared important but significantly lower than that of ascorbic acid (0.87 ± 0.059) (*p* < 0.05). The effective concentration EC_50_ was 246.66 *μ*g/ml ± 100.16 and 130.71 *μ*g/mL ± 91.21 in *E. retusa* and ascorbic acid, respectively.

### 3.4. Pharmacological Activities *In Vivo*

#### 3.4.1. Acute Toxicity

The acute toxicity of *E. retusa* was conducted for evaluating their safety at different doses. *E. retusa* did not generate any behavioral changes, and no death was observed with the maximum dose of 1000 mg/kg BW. In this study, a dose of 200 mg/kg BW was chosen to investigate the antioxidant, analgesic, and anti-inflammatory activities of the methanolic extract of *E. retusa* in experimental animals.

#### 3.4.2. Writhing Test

Table 3 shows acetic acid-induced writhing responses in mice. The methanolic extract of *E. retusa* (200 mg/kg) and paracetamol (100 mg/kg) significantly reduced the number of abdominal writhings elicited by acetic acid when compared to the control group, indicating their analgesic activities. The percentages of inhibition of the pain were 55% for the extract of *Euphorbia* and 70% for the paracetamol.

#### 3.4.3. Hot-Plate Test

The results obtained from the hot-plate test are presented in Table 4. The oral pretreatment of mice with the *E. retusa* methanol extract caused a significant (*p* < 0.001) increase of the latency time of reaction in comparison with the control group. The reference drug significantly inhibited the hot-plate-induced pain compared to the control group. The inhibition rates of hot-plate-induced pain by *E. retusa* compared with the positive control were 78.90% and 75.01%, respectively.

#### 3.4.4. Anti-Inflammatory Activity

**Figure 4:**
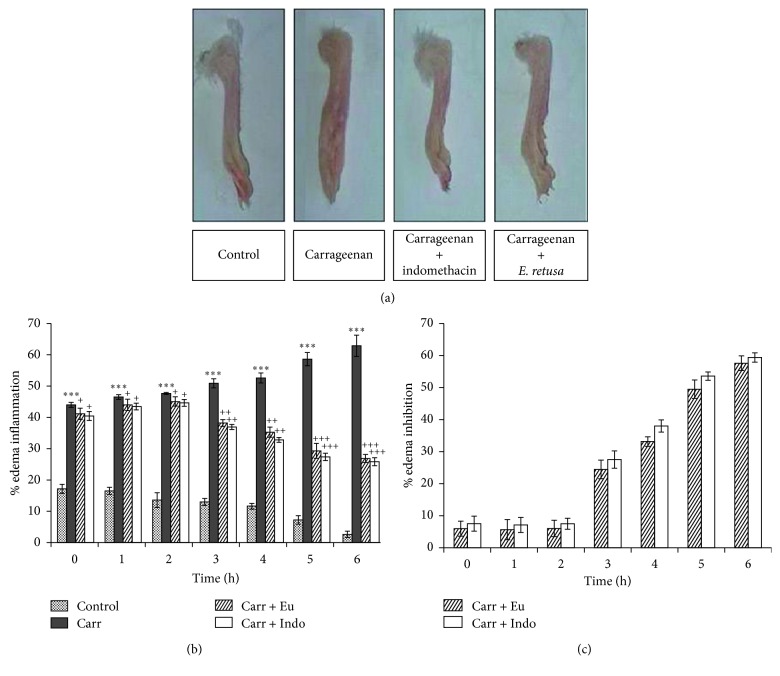
(a) The paw of mice treated with carrageenan and/or indomethacin or the methanolic extract of *Euphorbia retusa*. (b) Percentage inflammation of the paws of mice induced by local injection of carrageenan and/or indomethacin or the methanolic extract of *Euphorbia retusa*. Control: normal mice injected with isotonic saline solution (NaCl) 0.9%; Carr: mice injected with carrageenan 1%; Carr + Indo: mice pretreated with indomethacin and injected with carrageenan; Carr + Eu: mice pretreated with the methanolic extract of *E. retusa* and injected with carrageenan. (c) Percent inhibition of paw edema in mice pretreated with the *Euphorbia retusa* extract and indomethacin, induced after local injection of carrageenan. Values are represented as mean ± SD (*n*=6) in each group. ^*∗*^*p* < 0.05, ^*∗∗*^*p* < 0.01, and  ^*∗∗∗*^p  < 0.001. ^*∗*^*p*: compared with the control; ^+^*p*: compared with carrageenan.

The development of edema in control and treated groups for 6h is represented in Figure 4. The percentage of inflammation in the group (Carr) was increased progressively, and in a highly significant (*p* < 0.001) way over time, it was increased from 65% at the first hour to 120% at the 6th hour. However, this percentage of inflammation was decreased over time and in a comparable manner in the groups pretreated with indomethacin or the methanolic extract of *Euphorbia*; in fact, it was decreased from 57% at the 2nd hour to 32% after 6 hours in the group pretreated with the extract of the plant (Carr + Eu). This is well confirmed in Figure 4(a) that presents the paws of the different groups of mice taken after the completion of the experiment.

As seen in Figure 4(c), the extract of *E. retusa* shows significant inhibition of edema in the 2nd, 3rd, 4th, 5th, and 6th hour after injection of Carr. However, this inhibition was more important than that of mice receiving Indo, used as positive controls indicating anti-inflammatory activities of *Euphorbia*.

**Figure 5:**
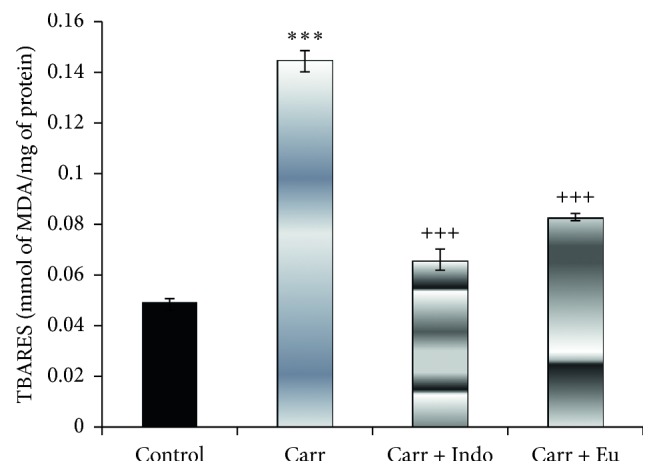
Effects of *Euphorbia retusa* and indomethacin on MDA concentrations in paw edema. The values are expressed as mean ± SEM (*n*=4;^*∗∗∗*^*p* < 0.001 compared with the control group; ^+++^*p* < 0.01 compared with the carrageenan-treated group).

*(1) Effects of the E. retusa Extract on the MDA Level*. The evolution of dermal lipid peroxidation is illustrated in Figure 5. In the Carr group, we noted a highly significant increase (*p* < 0.001) of the MDA level compared to the control group. However, the pretreatment of mice with the methanolic extract of *E. retusa* and indomethacin significantly decreased the MDA levels in paw tissue.

*(2) Effects of the E. retusa Extract on the Activities of Antioxidant Enzymes*. The changes of the activities of antioxidant enzymes in liver and paw tissues of all treated groups were assessed. The results presented in Table 5 show that carrageenan administration significantly decreased SOD, CAT, and GPx activities in liver and paw tissues in comparison with the control group (*p* < 0.001). However, a significant restoration in these enzymatic antioxidant activities was observed in the group pretreated with *E. retusa* (200 mg/kg), as well as with 10 mg/kg indomethacin.

## 4. Discussion

Nowadays, phytotherapy, based on scientific works, is becoming an important alternative pathway in the world to find effective natural medicines without any adverse effects. The objective of the present work is to study the phytochemical properties of the methanolic extract of *E. retusa* and to evaluate its antioxidant, analgesic, and anti-inflammatory activities. The phytochemical investigation of the methanolic extract of *E. retusa* revealed significant levels of polyphenols, tannins, and flavonoids. The HPLC analysis has provided more precise information on the chemical nature of the bioactive compounds present in the methanolic extract of *E. retusa*. Among the polyphenols, gallic acid, epicatechin, coumaric acid, apigenin, and naringenin and among the flavonoids, rutin, quercetin, and kaempferol were identified. Several studies have indicated that these phytochemical compounds are responsible for the antioxidant and anti-inflammatory properties of multiple medicinal plants [29, 30].

The antioxidant activity of the *E. retusa* methanol extract was evaluated *in vitro* by using the DPPH and FRAP tests. In methanol, DPPH occurs as a free radical of purple color that becomes weak after acquiring the proton of the antioxidant. Thus, measuring the reduction of the color intensity of the methanolic DPPH solution can be used to evaluate the ability of antioxidants to give the proton. The methanol extract exhibited good scavenging ability which may be due to donation of an electron or hydrogen to stabilize DPPH free radicals. In addition, in the present study, the *E. retusa* extract showed a powerful antioxidant activity on the FRAP assay which measures the ability to reduce the ferricyanide complex of Fe^3+^ to the ferrous (Fe^2+^) form through donating a hydrogen atom. Antioxidant potential of a plant extract might be due to its richness in polyphenols and flavonoids. These bioactive compounds are known by their redox properties and chemical structure, which might play an important role in chelating transition metals and scavenging free radicals [31].

Thermal stimulation by the hot plate and chemical irritation by acetic acid are the two analgesic testing methods employed with the objective of identifying possible peripheral and central effects of the *E. retusa* methanol extract. The acetic acid writhing test was used to study the peripheral analgesic effect of a substance. The methanol extract of *E. retusa* significantly reduced the number of writhings caused by the injection of acetic acid. The injection of acetic acid induces a response characterized by the contraction of the abdominal muscles, an extension of the forearms, and lengthening of the body. These symptoms are believed to be related to the release of endogenous mediators of pain such as serotonin, histamine, bradykinin, and prostaglandins. These chemical mediators stimulate peripheral nociceptive neurons and induce dilatation of arterioles and venules with contraction and separation of endothelial cells, resulting in increased vascular permeability [32]. The writhing test is a typical model to study the inflammatory pain. However, this test is very sensitive because the abdominal writhing response can be suppressed by muscle relaxants and nonsteroidal anti-inflammatory drugs (NSAIDs), such as aspirin, diclofenac, and indomethacin, which could lead to misinterpretation of the results. Therefore, it was considered that the use of other additional pain models is necessary. The hot-plate test was used to evaluate the central analgesic activity by measuring the reaction time of the perception of pain. The heat stimulation sensitizes peripheral nerve endings, and the impulses generated propagate to the brain via the spinal cord. Hence, this test is primarily used to evaluate the ability of a substance to inhibit pain of central origin. In our study, we found that *E. retusa* possess antinociceptive activity against chemically and thermally induced nociception. The mechanism of analgesic activity of *E. retusa* could be due to their bioactive substances that raised the pain threshold by depressing pain receptors centrally in the brain [33]. This antinociceptive activity may be related to the reduction in the liberation of the inflammatory mediators or to the blockage of receptors resulting in the peripheral antinociceptive effect [34]. The richness of our extract in phenolic compounds, mainly gallic acid, and flavonoids can justify this activity. These compounds are known by their anti-inflammatory activity because of their influence on the metabolism of arachidonic acid and the release of histamine; they inhibit the production of prostaglandin and the expression of cyclooxygenase [35]. Flavonoids also act on the expression of adhesion molecules and proinflammatory cytokines by various mechanisms including inhibition of transcription of the nuclear factor by inhibiting kinases involved in signal transduction [36].

In the present study, the anti-inflammatory activity of the methanolic extract of *E. retusa* was evaluated using the carrageenan-induced paw edema model. The molecular and cellular mechanism of the Carr-induced inflammation is well characterized and is widely used to study the inflammatory process of the skin, as well as to identify anti-inflammatory agents that may be useful in the treatment of local exogenous inflammatory disorders [37]. Carrageenan can induce local inflammation biphasic. The early phase (1-2 h) is related to the excessive production of chemical mediators such as histamine, serotonin, and bradykinin, while the delayed phase (3-6h) is linked to the production of prostaglandins, leukotrienes, and free radicals that contribute to the increase of vascular permeability, which facilitates the infiltration of neutrophils and accumulation of plasma fluid into the interstitial space which leads to edema [38]. The degree of paw swelling is a good index in assessing the anti-inflammatory activity. Our results revealed that the methanolic extract of *E. retusa* significantly inhibited the edema formation in both the first and second phases and in a manner similar to the indomethacin. The anti-inflammatory activity of *E. retusa* in the first phase could be due to the possible suppression of histamine signaling by the mast cell stabilizing effect [39] and direct inhibition of histamine H1 receptor and histidine decarboxylase gene transcriptions [40]. The anti-inflammatory activity of *E. retusa* increases in the second phase and reaches its maximum at 6 hours. This could be explained by the possible inhibition of the release and/or synthesis of cyclooxygenase (COX-2) or lipoxygenase products by the *E. retusa* extract. Indeed, the cyclooxygenase is an important enzyme that allows conversion of arachidonic acid to prostaglandins, under stimulation by various proinflammatory cytokines including bacterial lipopolysaccharide (LPS), TNF-*α*, IL-1*β*, IL-6, and interferon-*γ*, in the second inflammatory phase of the carrageenan-induced edema model [2]. Indomethacin (standard drug) also exhibited strong anti-inflammatory potential after 3, 4, and 5 h of carrageenan injection to mice. Nonsteroidal anti-inflammatory drugs (NSAIDs), such as indomethacin, target the cyclooxygenase enzyme, thereby inhibiting the formation of the paw edema [41]. Furthermore, the higher anti-inflammatory activity of the *E. retusa* extract seems to be closely correlated with its richness in polyphenolic constituents. In fact, flavonoids such as rutin, quercetin, and kaempferol, identified by HPLC in the methanolic extract of *E. retusa*, have been known in several previous studies as potent inhibitors of proinflammatory cytokines and cyclooxygenase [42]. These bioactif compounds can inhibit lysosomal enzyme secretion and arachidonic acid release from membranes by inhibiting lipoxygenase, cyclooxygenase, and phospholipase A2. From the cyclooxygenase pathway, arachidonic acid inhibition by inflamed cells could reduce endoperoxides, prostaglandins, prostacyclin, and thromboxanes, while from the lipoxygenase pathway, it could reduce hydroperoxy and hydroxyeicosatetraenoic acids and leukotrienes [43]. Then, anti-inflammatory activities of polyphenols have been well documented. In addition, several researches have reported that some polyphenols inhibit arachidonic acid peroxidation and possess cyclooxygenase inhibitory or stimulatory effects [44, 45].

The inflammatory response is also associated with the neutrophil infiltration and production of the reactive free radical species derived from them. In fact, inflammation induces excessive production of reactive oxygen species (ROS) involved in the genesis of oxidative stress, which generates oxidative imbalance [46]. Against oxidative stress condition, defense systems produce antioxidant enzymes, such as SOD, CAT, and GPx [47]. In the present work, we found that the carrageenan injection generates a highly significant decrease in the activities of SOD, CAT, and GPx in the untreated group at the tissue compared with the control group (*p* < 0.001). We assume that this reduction in activities may be related to the high use of the produced antioxidant enzymes that act as scavengers of free radicals generated during the inflammatory process. Hence, the oral pretreatment with *E. retusa* significantly increases the activity of enzymatic antioxidants (SOD, CAT, and GPx) compared with the carrageenan group. The anti-inflammatory effect exhibited by this plant might be related to its antioxidant properties [48]. During inflammatory processes, the ROS have been proposed to mediate cell damage via a number of independent mechanisms including the inactivation of antioxidant enzymes. Lipid oxidation serves as a marker of cellular damage *in vivo*. MDA production is due to free radical attack on the plasma membrane. Thus, the inflammatory effect would result in the accumulation of MDA. Enhancing the levels of glutathione and SOD then reduces the MDA production [49]. Therefore, endogenous glutathione plays an important role against carrageenan-induced local inflammation [50, 51]. In the present study, SOD, CAT, and GPx activities increased in the paw and liver tissues following the pretreatment with *E. retusa* and were coupled to the significant decrease in MDA. The antioxidant effects of *E. retusa* observed in our study *in vitro* and *in vivo* may be related to their richness in polyphenolic compounds. Among these compounds, gallic acids and flavonoids are capable of inhibiting the oxidants released by leukocytes and other phagocytes by increasing enzymatic antioxidants in tissues [52, 53]. These natural compounds, endowed with interesting antioxidant activities, could prevent multiple kinds of inflammations strongly linked to an excess of free radicals.

## 5. Conclusion

In conclusion, this work reveals the antioxidant, antinociceptive, and anti-inflammatory effects *in vitro* and *in vivo* of *E. retusa*. These effects seem to be related to their richness in polyphenolic compounds which inhibit the production of several inflammatory mediators such as MDA and COX-2 in the edema paw and increase the activities of SOD, CAT, and GPx in the liver. Thus, these bioactive compounds show an antinociceptive activity in both peripheral and central levels. Therefore, with these pharmacological properties, *E. retusa* can be considered an effective agent for the treatment of pain and inflammation.

## Figures and Tables

**Table 1:** The phytochemical composition and antioxidant capacity of the methanolic extract of *Euphorbia retusa*.

	Phytochemical composition			Antioxidant activity	
	Total phenolics (mg·GAE/g·DW)^a^	Total flavonoids (mg·QE/g·DW)^b^	Condensed tannins (mg·QE/g·DW)^c^	DPPH (EC_50_, mg/ml)	FRAP (EC_50_, mg/ml)
*Euphorbia retusa*	280.21 ± 0.085	20.50 ± 0.107	41.39 ± 0.198	131.23 ± 7.47	104.05 ± 12.82
Vitamin C	—	—	—	76.88 ± 16.88	48.46 ± 7.72

^a^Total phenolic content as the gallic acid equivalent. ^b^Total flavonoid content as the quercetin equivalent. ^c^Condensed tannin as the quercetin equivalent. Values are expressed as mean ± standard deviation (*n*=3).

**Table 2:** Composition of flavonoids and phenolic acids of the methanol extract of *Euphorbia retusa*.

	Compounds	Concentration (*µ*g/mL)
Phenolic acids	Gallic acid	1111.41
	Epicatechin	198.52
	Coumaric acid	147.73
	Apigenin	7.89
	Naringenin	6.13

Flavonoids	Rutin	50.74
	Quercetin	6.78
	Kaempferol	14.42

**Table 3:** Effect of the methanolic extract of *Euphorbia retusa* on the abdominal contortions induced by acetic acid in mice.

Group	Dose (mg/kg)	Number of writhes	Inhibition (%)
C (distilled water)	1 mL/kg	20.16 ± 8.97	—
*Euphorbia retusa*	200 mg/kg	9 ± 9.52^*∗∗*^	55.37
Paracetamol	100 mg/kg	6 ± 4.56^*∗∗∗*^	70.24

The values are expressed as mean ± SEM (*n*=6). ^*∗∗∗*^*p* < 0.001  and  ^*∗∗*^*p* < 0.01 as compared to the control.

**Table 4:** Effect of the methanolic extract of *Euphorbia retusa* on thermal nociception induced by a hot plate in mice paws.

Group	Dose (mg/kg)	Reaction time (s)	Inhibition (%)
C (NaCl)	10 mL/kg	31.85 ± 14.45	—
*Euphorbia retusa*	200 mg/kg	151 ± 27.69^*∗∗∗*^	78.90
Paracetamol	100 mg/kg	127.5 ± 34.06^*∗∗∗*^	75.01

The values are expressed as mean ± SEM (*n*=6). ^*∗∗∗*^*p* < 0.001 as compared to the control.

**Table 5:** Effect of carrageenan on antioxidant enzyme activities in liver and paw tissues of mice.

	Liver			Paw		
	SOD	CAT	GPx	SOD	CAT	GPx
Control	12.83 ± 2.67	1.94 ± 0.20	77.40 ± 4.63	20.08 ± 1.04	0.14 ± 0.01	70.32 ± 3.73
Carr	6.48 ± 0.28^*∗∗∗*^	0.53 ± 0.18^*∗∗*^	22.54 ± 4.92^*∗∗∗*^	8.66 ± 0.93^*∗∗∗*^	0.04 ± 0.01^*∗∗*^	12.23 ± 3.32^*∗∗∗*^
Carr + Indo	12.19 ± 0.35^+++^	1.45 ± 0.24^+^	54.74 ± 4.66^+++^	17.90 ± 1.23^+++^	0.12 ± 0.01^+^	44.75 ± 2.95^+++^
Carr + Eu	10.60 ± 0.52^+++^	0.89 ± 0.24^+^	45.35 ± 1.91^+++^	16.79 ± 1.06^+++^	0.10 ± 0.01^+^	26.73 ± 3.08^+++^

CAT: *µ*mol of H_2_O_2_ destroyed/min/mg of protein; SOD: U/mg protein; GPx: *µ*mol of NADPH oxidized/min/mg of protein. Values are expressed as mean ± SD of six mice in each group. Carr group versus control group: ^*∗∗*^*p* < 0.01  and  ^*∗∗∗*^*p* < 0.001. Carr + Indo or Carr + Eu group versus Carr group: ^+^*p* < 0.05, ^++^*p* < 0.01, and  ^+++^*p* < 0.001. Carr: carrageenan; Indo: Indomethacin; Eu: *Euphorbia retusa*.

## Data Availability

The data used to support the findings of this study are available from the corresponding author upon request.
